# Regulation of Epithelial-to-Mesenchymal Transition by Alternative Translation Initiation Mechanisms and Its Implications for Cancer Metastasis

**DOI:** 10.3390/ijms21114075

**Published:** 2020-06-07

**Authors:** Amit Bera, Stephen M. Lewis

**Affiliations:** 1 Atlantic Cancer Research Institute, Moncton, NB E1C 8X3, Canada; amitb@canceratl.ca; 2 Department of Chemistry & Biochemistry, Université de Moncton, Moncton, NB E1A 3E9, Canada; 3 Beatrice Hunter Cancer Research Institute, Halifax, NS B3H 4R2, Canada

**Keywords:** epithelial-to-mesenchymal transition (EMT), IRES, m^6^A-mediated translation, ITAF, cancer, metastasis

## Abstract

Translation initiation plays a critical role in the regulation of gene expression for development and disease conditions. During the processes of development and disease, cells select specific mRNAs to be translated by controlling the use of diverse translation initiation mechanisms. Cells often switch translation initiation from a cap-dependent to a cap-independent mechanism during epithelial-to-mesenchymal transition (EMT), a process that plays an important role in both development and disease. EMT is involved in tumor metastasis because it leads to cancer cell migration and invasion, and is also associated with chemoresistance. In this review we will provide an overview of both the internal ribosome entry site (IRES)-dependent and N^6^-methyladenosine (m^6^A)-mediated translation initiation mechanisms and discuss how cap-independent translation enables cells from primary epithelial tumors to achieve a motile mesenchymal-like phenotype, which in turn drives tumor metastasis.

## 1. Introduction

Protein synthesis is a complex, energy consuming biosynthetic process that comprises three different steps: initiation, elongation and termination [1,2,3,4]. It is well-documented that for eukaryotic protein synthesis the translation initiation step is rate-limiting and is tightly controlled, involving multiple eukaryotic initiation factors (eIFs; [4,5,6]). In general, there are two classes of translation initiation: canonical cap-dependent translation and non-canonical cap-independent translation initiation.

Over the past few decades the canonical cap-dependent translation initiation mechanism has been extensively studied [4]. Cap-dependent translation is a scanning mode of translation initiation in which a trimeric protein complex, known as eIF4F and consisting of eIF4E, eIF4A, and eIF4G, recognizes and binds to the 5ʹ-m^7^G cap structure of mRNA [3,4,7]. Following recruitment of other eIFs and the 40S ribosome, the translation initiation complex scans along the 5ʹ untranslated region (5ʹ-UTR) until it encounters the first initiation codon (AUG) in an appropriate context [3,4,7], where the 60S ribosome joins and polypeptide synthesis begins. In contrast, cap-independent translation does not typically require 5ʹ-end recognition or scanning; instead the 40S ribosome can directly bind in close vicinity to the start codon of the respective mRNAs [4,8]. Internal ribosome entry site (IRES)-mediated cap-independent translation is a well-characterized mechanism where the 40S ribosomal subunit is recruited by a subset of canonical eIFs and/or trans-acting factors to a position in close proximity to the initiation codon to mediate protein synthesis [8,9,10,11]. N^6^-methyladenosine (m^6^A) modification is the most abundant RNA modification and it has been recently shown that mRNAs containing m^6^A modification within their 5ʹ-UTRs are able to initiate translation in a cap-independent manner by binding to the eIF3 complex to recruit the 40S ribosome [12,13,14,15].

Under normal growth conditions cells and tissues use the cap-dependent translation mechanism for protein synthesis; however, under various stress conditions, such as nutritional stress, oxidative stress, and hypoxia, cap-dependent protein synthesis is significantly reduced [4,6,16]. Under these conditions cells are forced to synthesize stress-responsive proteins through alternative cap-independent translation mechanisms [17,18] for survival and recovery. In addition, cells also use the cap-independent protein synthesis machinery under multiple disease conditions such as neurodegenerative disease, auto-immune disease, and cancer [19,20,21,22].

Epithelial-to-mesenchymal transition (EMT) is an important event in embryonic development; the transition of epithelial cells to mesenchymal cells permits the formation of adult tissues and organs [23,24]. Although EMT plays an important role during embryonic development, it is also aberrantly activated during cancer metastasis [24,25]. EMT allows cancer cells to acquire migratory and invasive phenotypes that lead to the dissemination of tumor cells throughout the body [26]. In addition, cancer cells that have undergone EMT have increased resistance to apoptosis, oncogene-induced senescence and exhibit increased resistance to chemotherapy [27,28]. EMT is modulated at different levels of control, such as transcriptional control, epigenetic modifications, alternative splicing, and microRNA-mediated gene silencing. A group of transcription factors (Snail1, Snail2/Slug, Twist, and Zeb1/2) that simultaneously induce the expression of genes required for mesenchymal properties and repress the expression of genes that are required for the epithelial phenotype are a key factor in the regulation of EMT [29]. The expression of these EMT-regulating transcription factors can in turn be controlled at the transcriptional level by proteins such as NF-kB and both HIF-1α and HIF-2α [30,31], at the post-transcriptional level by microRNAs (miR-200 family; [32,33]), and at the post-translational level by proteasomal degradation [34]. EMT has also been shown to be tightly controlled at the translational level [35,36,37,38,39]; for example, Evdokimova et al. [35] reported that Y-box binding protein (YB-1) is a master regulator that mediates a switch from cap-dependent to cap-independent translation during EMT, thereby inducing selective translation of EMT-associated proteins that are necessary for the mesenchymal phenotype.

Given the emerging role for translational regulation of EMT-specific protein expression it is important to understand the mechanisms that contribute to such regulation in order to better understand how dysregulation of EMT is caused by altered translation initiation, and also how such dysregulation contributes to diseases such as cancer. Herein we will briefly describe the different mechanisms of cap-independent translation initiation and discuss their role for the regulation of EMT in cancer. 

## 2. EMT in Cancer

The process of cell de-differentiation via EMT is activated in many cancer cells and is currently accepted as one of the hallmarks of cancer [26,40,41]. Almost 80% of malignant tumors are derived from the epithelial tissues of different organs such as the lung, colon, breast, pancreas, prostate, bladder, ovary, kidney, and liver [42]. Moreover, cancer cells in early tumor states remain epithelial and have cohesive cell–cell junctions that inhibit their movements, and therefore they do not have migratory and/or invasive properties [43]. Upon overexpression of mesenchymal specific factors, including fibronectin, vimentin, or neural cadherin (N-cadherin), the epithelial tumor cells exhibit mesenchymal features, such as mobility and invasion [44,45]. The acquisition of these mesenchymal features can be explained at the mechanistic level by the activation of EMT, which is frequently dormant in early tumor cells [26]. Upon EMT activation, cancer cells dissociate from the primary tumor and undergo intravasation into blood vessels [26], where they can migrate far from the primary site to establish a distant metastatic tumor. The development of metastasis is responsible for approximately 90% of cancer mortality [46].

Investigations utilizing a number of loss-of-function and gain-of-function xenograft tumor models described the link between the activation of EMT and the degree of malignancy of a tumor [47,48,49]; however, metastasis formation may not be directly correlated with tumor size, as a recent report shows that tumor cell migration and micro-metastases can be found in early stages of cancer [50]. Activation of EMT in cancer cells leads to the activation of genes that regulate cell differentiation, proliferation, anti-apoptotic responses, epithelial and mesenchymal cell markers, proteolytic digestion of the cell–cell junctions receptors, activity of adhesion molecules that assist in cell movement, and the activation of extracellular matrix (ECM)-degrading proteases on the cell surface [51]. EMT in cancer cells follows the same pathways as normal physiological EMT, such as loss of epithelial cell polarity that leads to a disruption of cell–cell adhesion, cytoskeleton reorganization by release of mesenchymal-specific matrix metalloproteases (MMPs), and degradation of the ECM [52,53]. Moreover, stromal cells produce additional MMPs to increase the degradation of ECM and promote invasion [54]. In addition, to increase cell mobility the MMPs also generate extracellular E-cadherin fragments through proteolytic cleavage of E-cadherin [55]. Altogether, the changes induced by EMT promote the migration of cancer cells to establish metastatic tumor sites.

Current evidence suggests that activation of EMT also contributes to chemotherapy resistance in multiple cancer types and therefore EMT may serve as a potential target for overcoming chemoresistance. After chemotherapy, a significant increase in the expression of mesenchymal markers has been detected in breast, colorectal, and non-small cell lung cancers [56,57,58]. Earlier studies showed the connection between EMT and drug resistance by assessing the drug sensitivity of cancer cell lines with altered expression of EMT-specific transcription factors [27,59]. In line with these observations, using genetically-engineered mouse models it was demonstrated that inhibition of EMT-specific transcription factors and post-transcriptional regulators of EMT abrogates EMT-induced chemoresistance in breast and pancreatic cancer models [60,61]. These studies provide strong evidence linking EMT to chemoresistance and highlight the potential of targeting EMT for cancer therapy. 

The cellular trans-differentiation from epithelial to mesenchymal states is regulated by many signaling pathways, of which the Ras-ERK, MAPK and TGF-β pathways are among the best characterized. These pathways trigger the activation of key transcription factors that serve as master regulators of cell–cell adhesion, cell polarity, and motility. Major EMT-inducing transcription factors such as the zinc-finger-binding transcription factors Snail1 and Snail2/Slug, the basic helix-loop-helix (bHLH) factors TWIST1 and TWIST2, and the zinc-finger E-box-binding homeobox factors ZEB1 and ZEB2, mainly repress the genes associated with the epithelial phenotype and induce the expression of mesenchymal genes, ultimately leading to the cellular hallmarks of EMT [26]. In addition to their regulation by signaling pathways, master regulators of EMT are also significantly impacted by translational regulation. Enforced expression of YB-1 in non-invasive breast epithelial cells induces EMT and promotes metastasis by directly activating the cap-independent translation of Snail1 and other transcription factors implicated in the downregulation of epithelial and growth-related proteins and activation of mesenchymal proteins [35].

## 3. Cap-Independent Translation and EMT

It is well-accepted that the cap-dependent translation initiation mechanism is the primary means of protein synthesis under normal growth condition for eukaryotes, and this has been extensively reviewed elsewhere [2,3,4]; however, cap-dependent translation is inhibited during cellular stress, angiogenesis, neurodegenerative disease, viral infection, and EMT that occurs during development and metastasis [5,6,19,62]. It was initially unclear how some mRNAs are able to translate under these conditions, but in 1988 it was first reported that some viral mRNAs can bypass the cap-dependent translation initiation mechanism and use an alternate translation initiation pathway, namely cap-independent translation or IRES-mediated translation [63,64]. In addition, some mRNAs that do not contain an IRES are also able to translate under the conditions that enfeeble cap-dependent translation by utilizing N^6^-methyladenosine (m^6^A)-mediated translation initiation [12,13,14,15]. Each of these cap-independent translation initiation mechanisms have been implicated in the synthesis of proteins important for the control and/or maintenance of EMT, thereby exerting a level of regulation over the EMT process and its role in diseases such as cancer.

### 3.1. IRES-Mediated Translation Initiation and Its Regulation of EMT

In the late 1980s, two groups reported that poliovirus and encephalomyocarditis virus (EMCV) mRNAs can be translated in a cap-independent manner where the 40S ribosome binds in close vicinity to the start codon [63,64]. The mRNA regions required for this direct recruitment of the 40S ribosomal subunit are known as IRES [63,64], and mRNAs that contain IRES within their 5ʹ-UTRs do not require eIF4E binding to the 5ʹ-cap structure to initiate translation. Moreover, since the 40S ribosome is directly recruited to the IRES, this recruitment may occur with or without the help of various canonical initiation factors [65,66]; indeed the requirement for canonical initiation factors in IRES-mediated translation initiation is a variable factor for IRES in different mRNAs. The well-characterized viral IRES possesses complex secondary and tertiary structures that aid the efficient binding of 40S ribosome [8,65] and in some cases there is no need for canonical translation factors. For many IRESs, a group of proteins known as IRES trans-acting factors (ITAFs) play an important role for IRES-mediated translation initiation [67]. These ITAFs are typically RNA-binding proteins that enhance or regulate cap-independent translation initiation and thereby ribosome recruitment, in addition to their other cellular functions.

**Figure 1:**
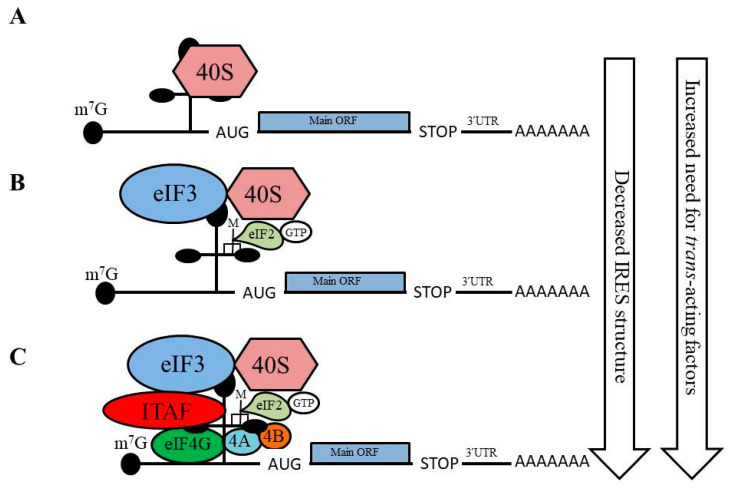
Overview of internal ribosome entry site (IRES)-mediated translation initiation. The requirement for canonical initiation factors and IRES trans-acting factors (ITAFs) varies among IRES-containing mRNAs. Several types of IRES-mediated translation initiation have been identified: (**A**) Group I: no canonical factors are required and the 40S ribosome is directly recruited to the mRNA by the IRES structure; (**B**) Group II: a few canonical initiation factors are required for 40S ribosome recruitment; (**C**) Groups III and IV: multiple canonical initiation factors, as well as ITAFs, are required for 40S ribosome recruitment and translation initiation.

There are four different groups of IRESs that have been classified based upon the requirement for initiation factors, the secondary structure of the IRES, and the position of the start codon relative to the IRES (Figure 1; [8,68]). Group I IRESs are highly-structured and compactly folded, which permits direct binding of the 40S ribosome without the assistance of any canonical initiation factors or Met-tRNA_i_ (Figure 1A; [69]). Group II IRESs exhibit to some extent the structure and folding of the IRES region as seen for Group I IRES; however, the Group II IRESs require some canonical initiation factors along with Met-tRNA_i_ to recruit the 40S ribosome (Figure 1B; [70]). Group III and IV IRESs are not properly structured and have a highly flexible configuration; along with canonical initiation factors and Met-tRNA_i_ Group III and IV IRES also require ITAFs for proper recruitment and binding of the 40S ribosome (Figure 1C). Although the Group III and IV IRESs require the same accessory factors to mediate 40S ribosome recruitment, the main difference between these two groups is the requirement for 40S ribosome scanning to initiate translation. For Group III IRESs, 40S ribosome scanning is unnecessary, as these IRESs initiate translation at the 40S ribosome recruitment site [71]. In contrast, for Group IV IRES the 40S ribosome scans the untranslated region in a 5ʹ to 3ʹ direction to reach a downstream AUG start site to facilitate translation initiation [70,72]. In general, the highly-structured and compactly folded Group I and II IRESs require fewer canonical initiation factors and ITAFs to initiate translation than the less structured Group III and IV IRESs.

In addition to viral mRNAs, several eukaryotic mRNAs use IRES for their translation under various stress conditions, during angiogenesis, or during EMT that occurs in development and cancer [73]. Macejak and Sarnow first reported a cellular IRES in the mRNA that encodes the immunoglobulin heavy-chain binding protein (BiP; [74]). Following this discovery several cellular mRNAs that contain an IRES have been reported and current estimates suggest that almost 10–15% of cellular mRNAs could be translated through an IRES-dependent translation mechanism [73,75]. A comparative screen by Weingarten-Gabbay et al. recently reported that ~10% of human mRNAs can be translated by cap-independent IRES-mediated translation [76]. The IRESite database contains a list of the many viral and cellular IRES-containing mRNAs and the list is continuously growing [77].

Cellular IRES are typically found in mRNAs that encode proteins involved in the regulation of cellular differentiation, cell cycle progression, apoptosis, stress, and EMT [73,78,79,80,81,82,83]. Moreover, it has been shown that different cellular IRESs have varying levels of activation in response to the conditions that reduce cap-dependent translation [84]. Multiple studies have shown that the differential response of IRES activity to cellular conditions is regulated largely by ITAFs [85,86]. A striking feature of many ITAFs is that they belong to a group of heterogeneous nuclear ribonucleoproteins, known as hnRNPs (e.g., hnRNP A1, C1/C2, I, E1/E2, K and L), which are RNA-binding proteins that shuttle between the nucleus and the cytoplasm [67,85,86]. Moreover, the hnRNP proteins are also involved in the regulation of mRNA splicing, stability, and transport [87], suggesting crosstalk between translation and RNA processing. Although ITAFs are generally believed to be able to increase or decrease (in rare cases) the affinity of binding between IRES and other translation initiation factors, the exact mechanism(s) by which ITAFs regulate IRES-mediated translation is largely unknown. It has been shown that: (1) ITAFs can act as chaperones to remodel IRES spatial structures to create conformations with higher or lower affinity for components of the translation apparatus [67,85,88]; (2) ITAFs function as a adaptor proteins that create or destroy bridges between the mRNA and the ribosome, in addition to those provided by canonical initiation factors [67,85,88]; and (3) ITAFs can take the place of canonical initiation factors in bridging an interaction between the mRNA and the ribosome [67,85,88]. In vitro assays show that without the addition of ITAFs, the activity of most IRES is fairly weak; ITAF addition has been shown to increase the translation efficiency of several IRESs [89]. Hence, the efficiency of IRES-mediated translation varies in different cell types and/or physiological conditions depending on the expression level of ITAFs.

Although it is clear that the availability of ITAFs plays an important role in modulating the activity of IRES, the mechanism(s) that regulate ITAF activity have not been well-defined. Several studies have suggested that the subcellular distribution of ITAFs (nuclear or cytoplasmic) is an important determinant of IRES activity [85]. There are two hypotheses to explain the effects of ITAF localization: (1) the nuclear-localized ITAFs may sequester IRES-containing mRNAs in the nucleus by binding to the IRES, thereby preventing access to the translational machinery [90]; or (2) ITAFs themselves are sequestered in the nucleus, thereby separated from their target IRES-containing mRNAs present in the cytoplasm. Under the appropriate signals (caused by stress or other physiological conditions), either the ITAF-bound mRNAs (in the first model) or the ITAFs themselves (in the second model) translocate from the nucleus to the cytoplasm, allowing translation of the IRES-containing mRNAs to proceed [85].

The Y-box binding protein (YB-1), a DNA/RNA-binding protein with a conserved cold-shock domain, is associated with cancer aggressiveness [91,92]; however, the exact role of YB-1 in cancer is unclear and has different effects depending on its subcellular localization. YB-1 behaves as oncogene in the nucleus, where it acts as a transcriptional activator by binding to Y-box elements in the promoter regions of pro-growth genes to induce proliferation [91]. In contrast, YB-1 acts as tumor suppressor in the cytoplasm where it can inhibit cap-dependent translation of pro-growth genes by binding at 5ʹ-UTRs to restrict binding of eIF4E to the 5ʹ-m^7^G cap [91]. Evdokimova et al. first reported that YB-1 regulates EMT through a novel mechanism involving the cap-independent translation of mRNAs that encode EMT regulators in Ras-transformed cells [35]. They found that YB-1 is capable of inducing an EMT-like morphological change in H-Ras-transformed human MCF-10A (MCF10-AT) and rodent EpH4 mammary epithelial cells, as well as in various carcinoma cell lines, including the cervical HeLa cell line and the prostate PC3 cell line. In addition, microarray analysis revealed that translation of >80% of mRNAs is negatively affected by YB-1 expression, but a small subset of mRNAs shows an increase in translation. These include mRNAs encoding transcriptional inducers of EMT and metastasis, such as Snail1, Zeb2/Sip1, HIF-1α, Lef-1 and TCF4. The 5ʹ-UTR of Snail1 mRNA is predicted to form a highly stable GC-rich stem-loop structure, similar to the structure of many IRESs; indeed, cap-independent translation initiation of Snail1 was found to be activated by YB-1 binding to the Snail1 5ʹ-UTR [35]. In parallel, YB-1 overexpression inhibited cap-dependent translation of proteins involved in cell proliferation, thereby inducing proliferation arrest in Ras-transformed human breast epithelial cells. These observations suggest the critical role of YB-1 in the coordinated induction of EMT through IRES-mediated Snail1 translation and proliferation arrest [93,94].

Shortly thereafter our laboratory reported that decreased expression of the translation initiation factor eIF3e induces EMT in breast epithelial cells [23]. Like YB-1, previous studies had reported that eIF3e can act as either a tumor suppressor [95,96] or an oncogene [97] in breast cells. We showed that decreased expression of eIF3e in the breast epithelial cell lines MCF-10A and MCF-12A leads to EMT, which in turn imparts invasive and migratory properties to breast epithelial cells. We found that a decrease in eIF3e expression causes a loss of expression of the epithelial markers E-cadherin and ZO-1 and an increase in the expression of the mesenchymal markers N-cadherin and vimentin. In addition, a decrease in eIF3e expression causes reduction of proliferation and enhanced migration of breast epithelial cells, phenotypes that are typical of cells that have undergone EMT [98]. Moreover, we reported reduction of eIF3e causes a decrease in global, cap-dependent translation; however, we also observed an increase in the abundance of the EMT regulators Snail1 and Zeb2. The increase in Snail1 and Zeb2 expression is due to preferential translation of Snail1 and Zeb2 mRNAs in cells that have reduced eIF3e expression. In a follow-up study we showed that reduced expression of eIF3e also induces EMT in lung epithelial cells (A549; [99]). Like breast epithelial cells, in lung epithelial cells a decrease in eIF3e expression causes loss of expression of the epithelial markers E-cadherin, increased expression of the mesenchymal markers N-cadherin and vimentin, reduced proliferation and enhanced migration.

We have found that in addition to inducing EMT, a reduction in eIF3e expression also causes a repression in global cap-dependent translation while simultaneously favoring the cap-independent translation (such as IRES-mediated translation) of mRNAs that rely on alternative translation initiation mechanisms, which are similar to the observations of Chiluiza et al. for the effects of a mutant form of eIF3e [100]. Chiluiza et al. reported that expression of a truncated version of eIF3e, similar to an isoform previously observed in breast tumor cells [100], causes a shift from cap-dependent to cap-independent IRES-mediated translation [100]. Using polysome profiling we examined the translation of mRNAs that encode proteins that regulate EMT, such as Snail1, ZEB2, Vimentin and Slug, in eIF3e-deficient A549 cells and found that ZEB2 and Vimentin translation is increased, whereas translation of Snail1 and Slug is maintained despite a decrease in global, cap-dependent translation [99]. Interestingly, by performing a β-galactosidase/CAT bicistronic reporter assay for IRES activity we found that the 5ʹ-UTRs of ZEB2, SNAI1, and vimentin exhibit IRES activity in A549 cells [99], suggesting that enhanced IRES-dependent translation of ZEB2, Snail1, and vimentin is responsible for their increased or maintained translation in eIF3e-deficient cells. Altogether, these observations indicate that upon reduced eIF3e expression in lung and breast epithelial cells ZEB2, Snail1, and vimentin rely on IRES-dependent translation for their expression and subsequent mediation of EMT [99].

Petz et al. reported that the enhanced expression of Laminin B1 (LamB1) during EMT correlates with elevated IRES activity [37]. Laminins constitute the extracellular matrix (ECM) as the main non-collagenous glycoproteins of the basement membrane and disrupt the migratory properties of cancer cells. LamB1 regulates the laminin-mediated integrin signaling that promotes cell adhesion, motility and differentiation [101] by acting as a ligand of the monomeric laminin receptor, which drives tumor cell invasion in hepatocellular carcinoma [102]. Petz et al. showed that LamB1 is translationally upregulated through IRES-mediated translation during EMT that occurs in hepatocellular carcinoma. In addition, they identified the La protein as an important ITAF that drives LamB1 translation. La is an RNA-binding protein involved in RNA processing and translation that predominantly localizes in the nucleus [102,103]. La had previously been shown to activate the IRES-dependent translation of XIAP, which supports tumorigenesis by preventing apoptosis [104]. During hepatocellular carcinoma EMT, La accumulates in the cytoplasm where it binds to the LamB1 IRES to enhance IRES-mediated translation [102]. This increased LamB1 translation results in upregulation of laminin-mediated integrin signaling through binding of LamB1 to the laminin and β1 integrin receptors, which were both found to be overexpressed in hepatocellular carcinoma [105,106], thereby leading to tumor cell invasion. Since LamB1 plays important role in the regulation of cell migration [107], increased IRES-dependent translation of LamB1 during EMT contributes to tumor cell migration and metastasis. Moreover, in a follow-up study it was shown that platelet-derived growth factor (PDGF) enhances the IRES activity of the LamB1 5ʹ-UTR by the increasing the cytoplasmic localization of La during EMT [108]. Together these findings suggest that regulated IRES-dependent translation of LamB1 is an important factor for the control of EMT in hepatocellular carcinoma.

### 3.2. m^6^A-mediated Translation and Its Regulation of EMT

**Figure 2:**
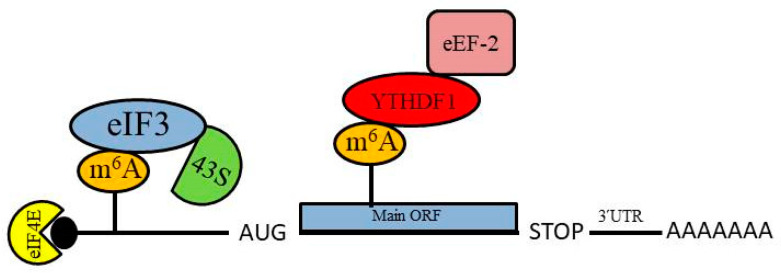
Overview of the role of N^6^-methyladenosine (m^6^A) modification of mRNA in protein synthesis. m^6^A modification of mRNA within the 5ʹ untranslated region (5ʹ-UTR) permits direct binding of the eIF3 complex to facilitate recruitment of the 43S pre-initiation complex, which mediates translation initiation. m^6^A modification within the coding sequence can enhance recruitment of the eEF-2 elongation factor via YDHTF1 binding to the m^6^A residue, which enhances translation of the mRNA.

Many eukaryotic mRNAs that contain N^6^-methyladenosine (m^6^A), a reversible base modification, have IRES-like activity that drives cap-independent translation initiation (Figure 2). m^6^A modification is the most abundant RNA modification, comprising roughly 80% of all RNA base modifications [109]. The functional effects of m^6^A modification on RNA are regulated by dynamic interactions among associated methyltransferases (“writers”), demethylases (“erasers”), and binding proteins (“readers”) [110,111]. Although m^6^A modification was first reported for the 3ʹ-UTRs of mRNAs, it can also occur within the coding region or the 5ʹ-UTR [13,112,113,114,115]. It was initially reported that m^6^A modification at the 3ʹ-UTR of mRNAs is responsible for the recruitment of RNA-binding proteins that affect mRNA stability [112]. In 2015, Meyer et al. reported that m^6^A modification at the 5ʹ-UTR of mRNAs plays a significant role for cap-independent translation initiation [13]; the m^6^A modification sites responsible for this cap-independent translation are referred to as ‘‘m^6^A-induced ribosome engagement sites’’ (MIRES). m^6^A-modified, uncapped mRNAs can be translated in cell-free extracts and capped, m^6^A-modified mRNAs can be translated in the absence of eIF4E, providing convincing evidence that m^6^A modification induces cap-independent translation initiation [13,116]. Moreover, m^6^A modification of the 5ʹ-UTR permits direct binding of the eIF3 complex, which is sufficient to recruit the 40S ribosomal subunit to initiate translation in the absence of the cap-binding protein eIF4E [13]. The m^6^A-mediated translation initiation mechanism was also shown to require 5ʹ-UTR scanning [13], and therefore resembles Group IV IRES-mediated translation, such as that observed for viral mRNAs that contain an eIF4G-binding IRES within their 5ʹ-UTRs [13,117,118].

It has been reported that during heat shock a single m^6^A modification within the 5ʹ-UTR of HSP70 mRNA enables cap-independent translation, providing a mechanism for selective mRNA translation under cellular stress [14]. Moreover, it is well-documented that HSP70 mRNA possesses an IRES within its 5ʹ-UTR [119]. At this point it remains unclear whether HSP70 utilizes both translation initiation mechanisms together or separately to increase cap-independent translation during heat shock. In addition, it is interesting to note that m^6^A modification of 5ʹ-UTRs is dynamic, and can be induced by UV irradiation, interferon-γ exposure, and heat shock [13,14]; however, in 2017, Ke et al. reported that m^6^A modification of mRNA occurs predominantly in the nucleus prior to mRNA splicing [120], suggesting that such regulation in response to stress would need to occur during mRNA biogenesis.

m^6^A modification of mRNA has been found to be directly associated with EMT and several reports suggest that m^6^A modification plays a multi-functional role in TGF-β expression and EMT regulation [121,122,123]. The functional effects of m^6^A on mRNA are regulated by dynamic interactions among (1) methyltransferases, such as catalytic subunit methyltransferase like 3 (METTL3) and its cofactor (METTL14) (“writers”); (2) demethylases, such as FTO and ALKBH5 (“erasers”); and (3) binding proteins, such as YTHDF family proteins, eIF3 complex members, and IGF2BP family proteins (“readers”) [110,111]. Li et al. reported that TGF-β-induced EMT is inhibited in cells that have reduced expression of METTL3 [122], a “writer” that methylates adenosines, suggesting that m^6^A modification of mRNA is important for the EMT process. They first demonstrated that m^6^A modification negatively regulates the levels of TGF-β1 protein, as METTL3 is required for the m^6^A modification of both TGF-β1 pre-mRNA and mature mRNA that in turn results in decreased TGF-β1 protein expression. In addition, METTL3 modulates the secretion and activation of TGF-β1 by disrupting TGF-β dimer formation [122]. These findings initially suggested that METTL3 expression, and therefore m^6^A modification of mRNA, has a negative effect on TGF-β1-induced EMT; however, Lin et al. further found that reduced METTL3 expression downregulates Snail1, a master regulator of EMT, which results in an inhibition of TGF-β-induced EMT [124]. This finding demonstrates that m^6^A modification of SNAI1 mRNA plays an important role in the regulation Snail1 expression, and that this regulation of Snail1 expression by m^6^A modification is a key factor in the progression of TGF-β1-induced EMT. Moreover, this observation raised the possibility that m^6^A modification of SNAI1 mRNA may regulate Snail1 abundance by affecting its translation, thereby controlling its activity during EMT. These findings suggest that m^6^A modification of mRNA is important for regulating the EMT process, and that this occurs by modulating the abundance of proteins that are important for EMT.

Overall m^6^A modification of mRNA was found to increase during EMT, including the m^6^A modification of SNAI1 mRNA [124]. Sequence analysis to detect m^6^A modification of mRNA on a global scale identified a ≥1.5-fold increase in the m^6^A modification of 128 mRNAs during EMT, several of which were found by gene ontology analysis to encode proteins related to cell migration and adherens junctions [124]. Moreover, this study found that the EMT-specific transcription factor Snail1 is affected by m^6^A modification during EMT. SNAI1 mRNA was found to be m^6^A modified within its coding sequence (CDS) and 3ʹ-UTR regions and showed a significant 2.3-fold enrichment in m^6^A modification during EMT progression. Gain- and loss-of-function experiments suggest that m^6^A modification within the SNAI1 CDS can enhance Snail1 translation elongation via interaction with YTHDF1, a “reader” that recognizes m^6^A-modified mRNA, which promotes the recruitment of the eEF-2 translation elongation factor and thereby enhances Snail1 translation [124]. Together, these findings demonstrate that m^6^A modification of mRNA can regulate the progression of EMT by affecting the translation of Snail1.

It is still early days for the exploration of the role of m^6^A modification of mRNA in the regulation of EMT. Given that there are at least 128 mRNAs whose m^6^A modification is increased during EMT [124], and that m^6^A modification has been shown to mediate cap-independent translation [13], it will be interesting to explore the effects m^6^A-mediated translation on the expression of proteins that are important for EMT. It is highly likely that the cap-independent translation of these EMT-specific proteins is mediated by m^6^A modification, thereby conferring a further layer of control of EMT through regulated protein synthesis. Moreover, the eIF3 complex is responsible for m^6^A-mediated translation initiation [13] and components of the eIF3 complex, such as eIF3e, have been shown to control EMT by affecting protein synthesis [23,99]. Together these findings raise the intriguing possibility that m^6^A-mediated translation initiation and changes to the eIF3 complex work in tandem to regulate translation initiation during EMT.

## 4. Conclusions

**Figure 3:**
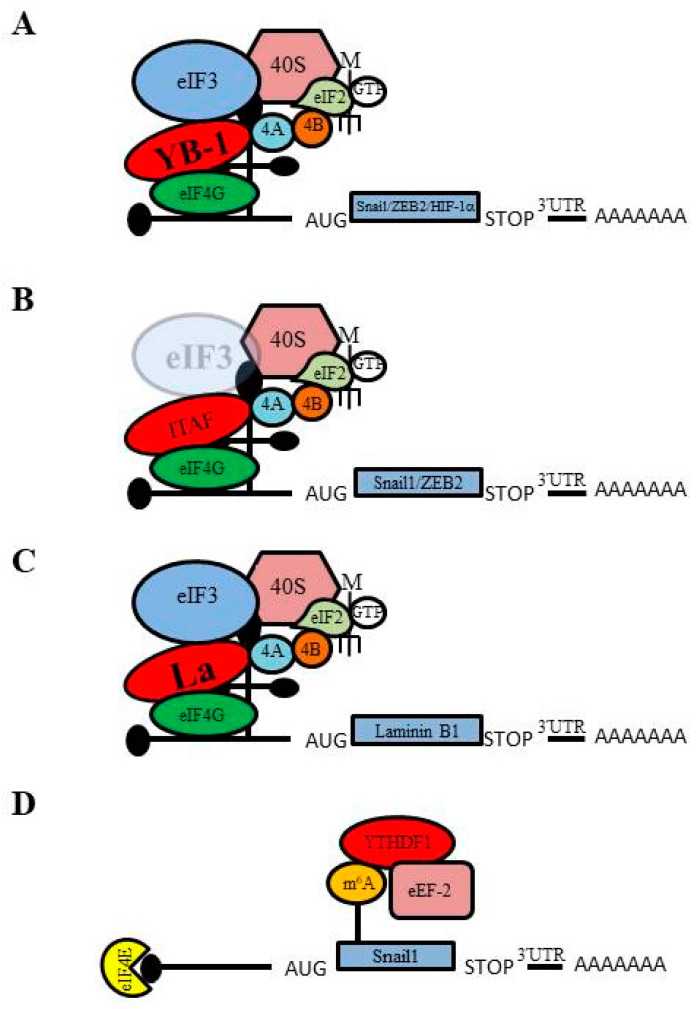
Alternative translation mechanisms are involved in the synthesis of proteins with roles in epithelial-to-mesenchymal transition (EMT). (**A**) The YB-1 protein recruits the translation initiation machinery to mRNAs that encode Snail1, ZEB2, and HIF-1α to support their synthesis, leading to EMT. (**B**) Reduced expression of the initiation factor eIF3e facilitates the translation of the Snail1 and ZEB2 proteins to mediate EMT. (**C**) The ITAF La mediates the translation of Laminin B1 to drive cells toward EMT. (**D**) m^6^A modifications within the Snail1 open reading frame permit enhanced recruitment of the elongation factor eEF-2 via YTHDF1 to increase Snail 1 translation and facilitate EMT.

EMT is essential for normal embryogenesis, but also promotes cancer progression, metastasis, and chemoresistance. A complex network of transcriptional regulators, epigenetic modifications, microRNA regulators, and splicing mechanisms control the execution of EMT, and a wide variety of signaling pathways have been reported to activate EMT in various normal and cancerous tissues. Moreover, it has recently emerged that cancer cells can undergo EMT by activating alternative modes of mRNA translation initiation, such as cap-independent translation, to support the synthesis of proteins with important roles in EMT (Figure 3). Although several reports have implicated IRES-mediated translation and m^6^A modification of mRNA in the regulation of EMT, further investigation is necessary to obtain a detailed understanding of how these translation initiation mechanisms affect and/or control EMT. This knowledge will aid in the development of novel therapeutics that target EMT and thereby metastatic cancer and chemoresistance.
